# Treatment of Extensively Drug-Resistant (XDR) *Acinetobacter* in US Veterans’ Affairs (VA) Medical Centers

**DOI:** 10.1017/ash.2021.138

**Published:** 2021-07-29

**Authors:** Margaret Fitzpatrick, Katie Suda, Linda Poggensee, Amanda Vivo, Geneva Wilson, Makoto Jones, Martin Evans, Nasia Safdar, Charlesnika Evans

## Abstract

**Background:** Infections caused by *Acinetobacter* spp are often healthcare acquired and associated with high mortality. Extensively drug-resistant (XDR) *Acinetobacter* are nonsusceptible to at least 1 agent in all but 2 or fewer antibiotic classes. Few of the new antibiotics targeting multidrug-resistant gram-negative bacteria are effective against XDR *Acinetobacter*. Recent national guidelines for treatment of resistant gram-negative infections do not include *Acinetobacter*, leaving a knowledge gap in best practices. **Methods:** This retrospective cohort study included microbiology, clinical, and pharmacy data from all patients hospitalized between 2012 and 2018 at any Veterans’ Affairs medical center who had cultures that grew XDR *Acinetobacter* spp. Bivariate unadjusted analyses compared clinical outcomes by monotherapy versus combination therapy. Using mixed-effects ordinal logistic regression, propensity score–adjusted models accounting for severity of illness and other variables associated with treatment were fit to compare outcomes. **Results:** Of 11,546 patients with 15,364 cultures that grew *Acinetobacter* spp, 408 patients (3.5%) had 666 cultures (4.3%) with XDR *Acinetobacter*. Moreover, 276 of these patients (67.6%) had gram-negative targeted antibiotic treatment within −2 to +5 days from the culture. Furthermore, 118 patients (42.8%) received monotherapy, most commonly piperacillin-tazobactam (n = 54, 45.7%) or an anti-*Pseudomonas* cephalosporin (n = 21, 17.8%). Also, 158 (57.2%) patients received combination therapy, most commonly a carbapenem (n = 93, 58.9%) and/or polymyxin (n = 68, 43.0%). Moreover, 41 patients (25.9%) received both a carbapenem and polymyxin. In both unadjusted and adjusted analyses, there were no significant differences in the odds of 30-day mortality (aOR, 1.43; 95% CI, 0.86–2.38) or 1-year mortality (aOR, 1.04; 95% CI, 0.68–1.60) between combination therapy and monotherapy groups. Among 264 patients (96%) whose cultures occurred during an inpatient or long-term care admission, unadjusted analyses showed increased odds of in-hospital mortality (OR, 1.89; 95% CI, 1.08–3.29) and longer postculture length of stay in the combination therapy group: median, 23 days (IQR, 11–57) versus 14 days (IQR, 7–32) (*P* = .02). However, with propensity score adjustment, these associations were no longer significant. Furthermore, there was no significant difference in odds of 90-day readmission between groups in either unadjusted or adjusted analyses (aOR, 1.20; 95% CI, 0.74–1.95). **Conclusions:** In this large national cohort of patients with XDR *Acinetobacter* cultures, more patients received combination therapy than monotherapy, and carbapenems and polymyxins were the most-used classes. However, there were no significant differences in outcomes between patients receiving combination therapy and monotherapy, suggesting lack of clinical benefit to the common practice of treating XDR *Acinetobacter* infections with multiple antibiotics. Further research is needed to determine optimal treatment strategies for this pathogen.

**Funding:** No

**Disclosures:** None

